# Environmental sampling to assess the bioburden of *Mycobacterium avium* subspecies *paratuberculosis* in drylot pens on California dairies

**DOI:** 10.7717/peerj.8081

**Published:** 2019-11-19

**Authors:** Tapakorn Chamchoy, Deneice R. Williams, John M. Adaska, Randall J. Anderson, Sharif S. Aly

**Affiliations:** 1 Veterinary Medicine Teaching and Research Center, School of Veterinary Medicine, University of California, Davis, Tulare, CA, USA; 2 California Animal Health and Food Safety Laboratory, Tulare Branch, Tulare, CA, USA; 3 California Department of Food and Agriculture, Animal Health Branch, Sacramento, CA, USA; 4 Department of Population Health and Reproduction, School of Veterinary Medicine, University of California, Davis, CA, USA

**Keywords:** Environmental sampling, Quantitative real-time PCR, *Mycobacterium avium* subspecies paratuberculosis, Drylot pen, Intraclass correlation coefficient

## Abstract

*Mycobacterium avium* subspecies *paratuberculosis* (MAP) is a bacterium that can cause substantial economic losses in infected dairy herds due to reduced milk production and increased cow-replacement costs. In order to control MAP in dairies with drylot pens, a standardized environmental sampling protocol to quantify MAP in fecal slurry was developed based on an existing protocol for freestall pens. Specifically, following a 24 h hold of the flush, a grab sample of approximately 10 ml of fecal slurry was collected every 1 m along the flush lane of the drylot pens, avoiding individual cow fecal pats. To determine the reliability and repatability of the new environmental sampling protocol for estimation of MAP bioburden at the pen level, two collectors simultaneously collected fecal slurry samples every day for 3 days from six drylot cow pens on two Central California dairies. During the study period no cow movement between pens was allowed with the exception of sick cows. The study herds had MAP seroprevalence of 5.8% and 3.2%, respectively, based on whole pen serum ELISA results. Variance components models for quantitative real-time PCR (qPCR) results showed samples collected from different pens on different dairies accounted for greater variablitiy in MAP concentration (65%), while samples collected by different collectors had the least variability (0.1%). In contrast, variability in MAP concentration in environmental samples collected on different days had 25% variability. The intraclass correlation coefficient showed high reliability (93%) of environmental sampling simultaneously by different collectors. In contrast, the reliability of environmental sampling at different days was 65%, which was similar to the reliability for sampling by different collectors on different days. Investigators can expect high reliability when employing the new environmental sampling protocol along with qPCR testing of environmental samples from drylot pens.

## Introduction

*Mycobacterium avium* subspecies *paratuberculosis* (MAP) is an intracellular bacterium that causes a chronic granulomatous enteritis in ruminants commonly known as Johne’s disease. The clinical signs of MAP infected cattle are diarrhea, weight loss and edema due to hypoproteinemia caused by a protein-losing enteropathy (Sweeney et al., 2012). Johne’s disease can cause substantial economic losses in infected dairy herds due to reduced milk production (Aly et al., 2010) and increased cow-replacement costs (Smith, Al-Mamun & Gröhn, 2017). The US dairy industry losses up to $200 per cow in MAP test-positive herds compared to MAP test-negative herds (Ott, Wells & Wagner, 1999). In 1996, USDA’s National Animal Health Monitoring System estimated that Johne’s disease costs the US dairy industry $250 million annually (Ott, Wells & Wagner, 1999). Testing blood and fecal samples from individual cows for MAP can be time consuming and cost prohibitive in large dairy herds. In contrast, environmental samples offer a convenient, cost-effective alternative to identify MAP infected dairy herds (Berghaus et al., 2006; Aly et al., 2012). Environmental samples that can be tested for MAP include fecal slurry on freestall pen floors, boot swabs or a combination of both (Donat et al., 2016; Hahn et al., 2017).

When paired with quantitative real-time PCR (qPCR), environmental samples, specifically fecal slurry from dairy cow pens, have shown excellent reliability to quantify MAP bioburden in dairy herds with freestall pens (Aly et al., 2009). However, due to differences in pen design, the estimation of MAP bioburden in freestall pens does not directly apply to drylot pens. Freestall pens are more confined than drylot pens and have small, non-flushed cross-over alleys connecting two flush lanes, allowing for accumulation of feces representing the entire population in the pen. In contrast, drylot pens are large, open lots bedded with dirt and/or dried manure that have one flush lane (commonly known as the feed alley) located inside the pen parallel to the feed bunk. Fecal slurry commonly accumulates from all cows traveling along the flush lane in drylot pens. However, testing the fecal slurry from the flush lane in drylot pens has not been validated for MAP surveillance. Furthermore, the correlation of MAP bioburden in drylot pens and pen-level MAP shedding prevalence is also not known. Amongst the challenges that may have discouraged development of an environmental sampling protocol for drylot pens are the periodic flushing of the entire drylot pen alley once or more daily depending on the dairy’s management; and the lack of cross-over alleys that may serve as convenient, unflushed regions in the pen. The objective of this prospective longitudinal study was to estimate the reliability of environmental sampling on drylot pens between collectors and over time.

## Materials and Methods

### Study herds

A convenience sample of two Central Valley California dairy herds were identified and enrolled based on the willingness of the owners to participate in the study. The use of vertebrate animal for this study was approved by the Institutional Animal Care and Use Committee (University of California Davis Institutional Animal Care and Use Committee) with the approval reference number of 20986. In each herd, the cattle resting areas in the drylot pens were bedded with dried manure solids. Herd 1 was composed of 2,862 lactating Jersey cows housed in drylot pens. The flush lanes located adjacent to the feed lanes were flushed twice daily using recycled lagoon water with shade tarps over the flush lanes. The management of herd 1 routinely tested cattle at the cessation of milking at the end of lactation, commonly known as dry-off, for MAP antibodies using serum enzyme linked immunoassay (ELISA). Herd 1 had a MAP seroprevalence of 3.3% (95% CI [2.9–3.6]), based on ELISA testing of 8,354 serum samples between June, 2013 and December, 2017. Herd 2 was a mixed breed herd composed of 2,733 lactating Jerseys and 1,188 lactating Holsteins housed in drylot and freestall pens and was not routinely tested for MAP. Lactating cow pens, flush lanes were flushed five times while heifer and dry cow pens, flush lanes were flushed twice daily using recycled lagoon water and the latter had no shade structure or tarps over the flush lanes. Most of the lactating cows in herd 2 were housed in freestall pens with the exception of two drylot pens while all the dry cows were housed in drylot pens. Seroprevalence for MAP in herd 2 was unknown at time of enrollment.

### Study design

#### Prevalence estimation

Individual blood and fecal samples were collected from all the cows in the study pens (3 pens on each dairy, 6 pens total) to determine the prevalence of MAP prior to 3 days of environmental sampling for our reliability and repeatability study. On each dairy, no cows were moved to or from the study pens during the study period, except for sick cows moved to the hospital pen and such movements were documented. Blood samples for seroprevalence estimates were collected on November 13th and December 12th, 2017 from herd 1 and 2, respectively. Blood samples were taken from the coccygeal vein using 20 gauge vacutainer needles and serum tubes. Fecal samples were collected from the rectum using individual plastic sleeves for every cow, then transferred to two oz polypropylene tubes with attached lids. All samples were transferred on ice to the Dairy Epi lab (Aly Lab, Tulare, CA, USA). The blood samples in serum tubes were place upright in the rack at room temperature for at least 2 h from the collection to allow blood to clot. Blood samples were spun at 1 G for 5 min to harvest serum in the same day as collection. Serum samples were stored at 4 °C until testing by ELISA methods within 3 days of sample collection. Fecal samples from individual animals were stored at –80 °C until testing by qPCR.

#### Reliability and repeatability study

Both dairies’ herd managers were asked to stop flushing the study pens 24 h prior to the first day of environmental sampling. Environmental samples were collected at 24 h intervals for 3 days at approximately the same time of day by two sample collectors (study authors DW and TC). The flush lanes were flushed immediately following each sample collection on each day which allowed the fecal slurry to accumulate over the following 24 h and prior to the next sampling. Specifically, study pens on dairy 1 were sampled on November 14th–16th and on dairy 2 on December 13th–15th. Sample collector 1 had previous experience collecting environmental fecal samples for MAP testing. Sample collector 2 had no previous environmental sampling experience but had been trained prior to the study. Both collectors sampled fecal slurry from the same pens over the three study days to compare the similarity in MAP concentration in samples collected by different collectors (reliability) and on different days (repeatability). A grab sample of approximately 10 ml of fecal slurry was collected by hand with approximately every 1 m along the length of the flush lane, avoiding individual cow fecal pats. Disposable gloves were worn during the sampling and changed between each pen. Each collector collected fecal slurry into 2 L clean plastic containers. A clean container was used for each pen after being disinfected using Quaternary Ammonium Compounds, rinsed thoroughly with water and dried. After the container was filled with the fecal slurry from the entire pen’s flush lane, the slurry was mixed with a sterile wooden tongue depressor using 10 vertical stirs from the bottom to the top, followed by 10 clockwise, and finally 10 counterclockwise stirs. Approximately 25 ml of fecal slurry from each pen were then transferred to a 2 oz polypropylene tube with an attached lid which was then transported on ice to the laboratory. At the laboratory, each sample was split into 10 ml, stored in a smaller polypropylene wide opening screw cap container and the remaining volume stored into a conical polypropylene screw cap tube. All the fecal samples were stored at −80 °C until the entire study’s fecal sample repository was collected and ready for qPCR testing.

### Laboratory testing

#### ELISA

Serum samples from both dairies were tested using an ELISA designed to detect the presence of antibodies against MAP (MAP Antibody Test Kit, IDEXX, Westbrook, ME, USA). The test was performed according to the manufacturer specifications at a temperature between 18 and 26 °C. The absorbance was measured at 450 nm. The mean optical density (OD) of two negative and two positive controls were calculated. The mean OD for the two positive control wells needed to be more than or equal to 0.350 and the ratio of mean OD of the positive to negative controls needed to be more than or equal to 3.00 in order for the test results to be considered valid. The S/P ratio for each sample was calculated by the sample OD minus the negative control mean OD divided by the positive control mean OD minus the negative control mean OD. Negative, suspect and positive results were determined at S/P ratios ≤0.45, 0.46–0.54 and ≥0.55, respectively. Initially, samples were tested in single wells, however, any samples with positive or suspect results were re-tested in duplicate. The final results of these samples were based on the average S/P ratio from the duplicate test wells. The samples with suspect results were treated as positive results in our calculation.

#### qPCR

To estimate the prevalence of fecal shedding in each of the study pens a survey sample of cattle from each pen were identified and tested by qPCR. Specifically, fecal samples from all the seropositive and ELISA suspect cows were qPCR tested. In addition, fecal samples from a random sample of ELISA negative cows were also qPCR tested. Hence, environmental fecal samples, as well as the fecal samples from MAP ELISA positive and suspect cows and randomly selected fecal samples from ELISA negative cows were transported to CAHFS laboratory at University of California Davis on dry ice for qPCR testing. The MAP’s DNA extraction process was performed using MagMAX Total Nucleic Acid Isolation Kit with the requirement of at least 0.3 g of fecal sample. Subsequently, each DNA sample was tested a commercially available kit (VetMAX Gold MAP PCR Kit, Thermo Fisher Applied Biosystems, Waltham, MA, USA) on a Cepheid SmartCycler II according to the manufacturer’s instructions. Ct values were recorded up to 40 but a sample was only considered positive if the reaction crossed threshold at less than 37 cycles.

### Statistical analysis

#### Descriptive statistics

Descriptive statistics observed MAP seroprevalence and estimated MAP shedding prevalence were reported stratified by herd. The MAP shedding prevalence was based on qPCR and a survey sample, as described previously (Lavallée & Beaumont, 2015; Maier et al., 2019). Briefly, qPCR survey-adjusted prevalence results from seropositive or suspect cows were assigned a survey weight of one, while results from seronegative cows were assigned a survey weight that is the inverse of the sampling probability, with the sampling probability being the number of seronegative cows tested by qPCR in a pen divided by the number of seronegative cows in the pen.

#### Variance components models

**Figure 1:**
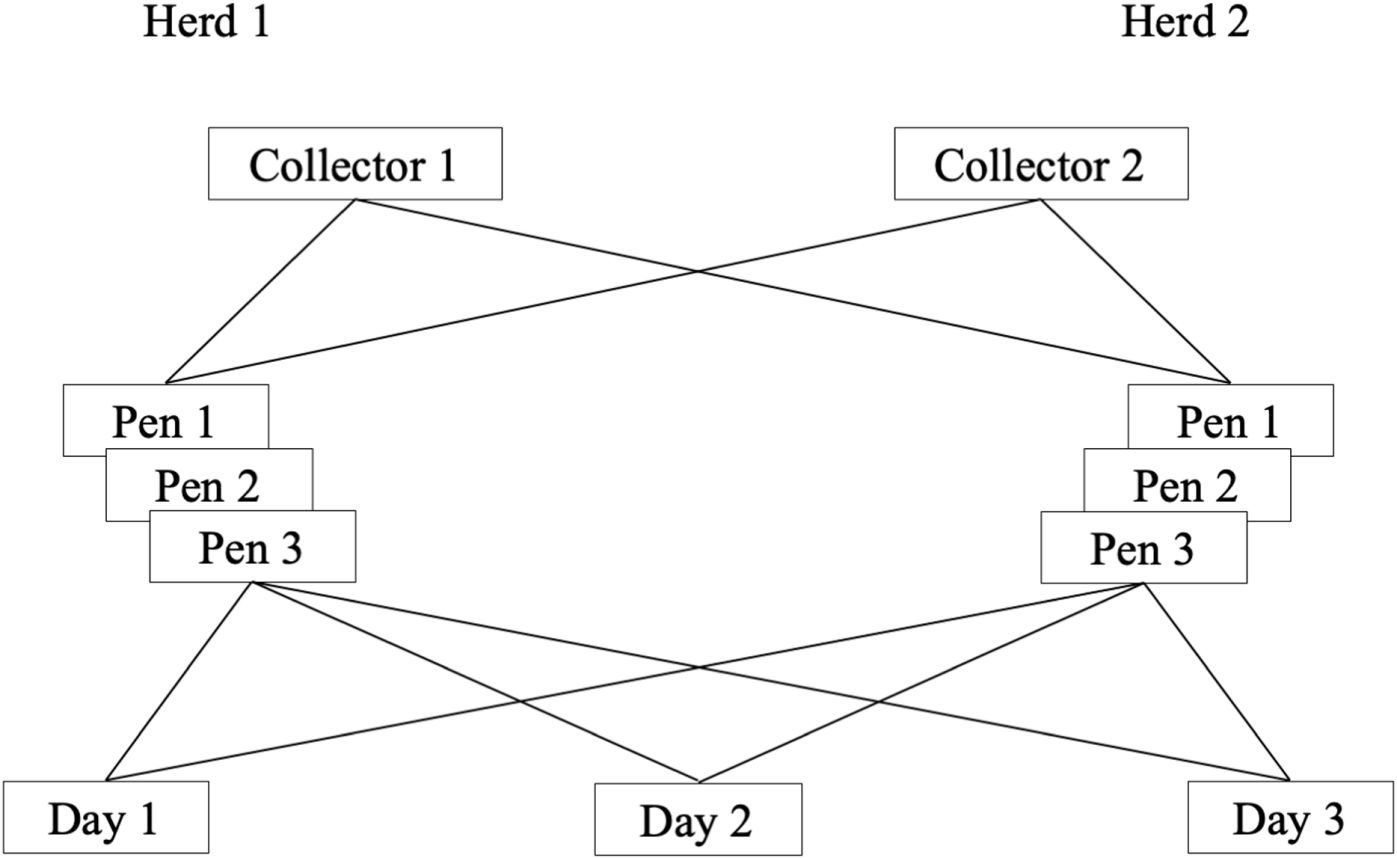
Schematic presentation of a longitudinal study of the reliability of environmental sampling and testing methods to quantify MAP^1^ in drylot cow pens in 2 California dairies. ^1^*Mycobacterium avium subsp. paratuberculosis*.

For each outcome, there were four factor levels: dairy, pen, collector and day (Fig. 1). Pens were nested within dairy. In dairy *i*, *i* = {1, 2} were pens *j*; where for both *i* = 1 and *i* = 2, *j* = {1, 2, 3}. Pens were cross classified by collector *k*; *k* = {1, 2}; and day *l*; *l* = {1, 2, 3}.

Variance components models with random intercepts for dairy *u_i_* and pen *v_ij_* were used to account for variability attributable to dairies and pens within dairies, respectively. The cross-classification of pens by collector and day was addressed by including random intercepts, *w_k_* for collector and *z_l_* for day (Goldstein, 1987; Marchenko, 2006). All random effects (*u_i_*,*v_ij_, w_k_, z_l_*) and residual error (*e_ijkl_*) were assumed to be normally distributed with mean = 0 and variances $\sigma _u^2,{\mkern 1mu} \sigma _v^2,\sigma _w^2,{\mkern 1mu} \sigma _z^2,{\mkern 1mu} {\sigma ^2}$, respectively. For each study outcome (*y_ijkl_*), cycles-to-threshold (CT) values, variance components were estimated from a multilevel mixed model (Eq. (1)).

(1) $${y_{ijkl}} = {\beta _0} + {u_i} + {v_{ij}} + {w_k} + {z_l} + {e_{ijkl}}$$

Models were fit using restricted maximum likelihood (Jiang, 2007). Quantile plots of model residuals and standardized residuals were evaluated for normality. Similarly, the empirical best linear unbiased predictions (EBLUP) for dairy, pen, collector and day from qPCR models were estimated and evaluated for normality.

Estimates of variability attributable to dairy, pen, collector and day as a percent of the total variability were computed for each test result. The intraclass correlation coefficient (ICC), an estimate of similarity in results of samples from the same cluster, was estimated for the following three ICC:

(2) $$(1)\quad {\rm{ICC}}({\rm{dairy}},{\rm{pen}},{\rm{day}}) = {{\sigma _u^2 + \sigma _v^2 + \sigma _z^2} \over {\sigma _u^2 + \sigma _v^2 + \sigma _w^2 + {\sigma ^2}}}$$

to estimate the similarity in MAP concentrations in environmental samples collected by different collectors (from the same dairy, pen and on the same day);

(3) $$(2)\quad {\rm{ICC}}({\rm{dairy}},{\rm{pen}},{\rm{collector}}) = {{\sigma _u^2 + \sigma _v^2 + \sigma _w^2} \over {\sigma _u^2 + \sigma _v^2 + \sigma _w^2 + \sigma _z^2 + {\sigma ^2}}}$$

to estimate the similarity in MAP concentrations in environmental samples collected on different days (from the same dairy, pen and by the same collector); and

(4) $$(3)\quad {\rm{ICC}}({\rm{dairy}},{\rm{pen}}) = {{\sigma _u^2 + \sigma _v^2} \over {\sigma _u^2 + \sigma _v^2 + \sigma _w^2 + \sigma _z^2 + {\sigma ^2}}}$$

to estimate the similarity in MAP concentrations in environmental samples collected by different collectors on different days (from the same dairy and pen). The 95% confidence intervals of the ICC were computed using the delta method. The Spearman rank correlation coefficient for MAP fecal shedding prevalence and MAP bioburden was estimated. The following ranges were used to interpret the correlation and ICC estimates: <40%, poor; 41–75%, fair to good; >75%, excellent (Fleiss, Levin & Paik, 2003). All analyses were performed using Stata (Stata/IC V. 15.0, College Station, TX, USA).

## Results

### Descriptive statistics

Samples collected from both herds were from Jerseys cows. For herd 1, pen 1 consisted of 193 lactating cows (<1% lactation 1, 41% lactation 2, 51% lactation 3 and 7% lactation 4 or higher). There were 260 lactating cows in pen 2 (10% lactation 1, 64% lactation 2, 19% lactation 3 and 6% lactation 4 or higher). For pen 3, there were 103 lactating cows (21% lactation 1, 7% lactation 2, 27% lactation 3 and 44% lactation 4 or higher). For herd 2, pen 1 consisted of 221 lactating cows (39% lactation 1, 46% lactation 2, 6% lactation 3 and 9% lactation 4 or higher). There were 31 dry cows in pen 2 (38% lactation 1, 28% lactation 2, 22% lactation 3 and 13% lactation 4 or higher). For pen 3, there were 320 dry cows (41% lactation 1, 26% lactation 2, 18% lactation 3 and 15% lactation 4 or higher).

During the study period, no cows were moved between the study pens on either dairies. The MAP prevalence based on ELISA and survey-adjusted qPCR results are summarized in Table 1. Table 2 shows the descriptive statistics for MAP qPCR results for the 36 environmental samples which ranged from 32.7 to 40.0 CT. A total of 26 out of 36 samples were positive and 10 out of 36 samples were negative for MAP using the manufacturer’s recommended cutoff of <37 CT. The target of the PCR kit used in this study is ISMap02. Previous work has shown that PCR reactions targeting this insertion sequence may detect other, non-MAP, species of Mycobacterium (Park et al., 2018).

**Table 1:** *Mycobacterium avium* subspecies *paratuberculosis* (MAP) prevalence based on all serum ELISA results and survey-adjusted qPCR results of stratified randomly selected fecal samples by pens.

	Prevalence	
	Seroprevalence (N, SEM)	qPCR Prevalence (N, SEM)
Herd 1	5.8% (556, 1.0%)	5.9% (89, 1.0%)
Herd 2	3.2% (572, 0.7%)	1.2% (59, 0.4%)

**Table 2:** Descriptive statistics for MAP^1^ concentration in environmental samples tested in duplicate. Samples were collected from drylot pens in two dairies by two collectors daily for 3 days.

Herd	Number of pens	Sample size	Day 1				Day 2				Day 3			
			Collector 1		Collector 2		Collector 1		Collector 2		Collector 1		Collector 2	
			Mean CT^2^	SD^3^	Mean CT	SD	Mean CT	SD	Mean CT	SD	Mean CT	SD	Mean CT	SD
1	3	556	36.7	3.2	37.1	2.6	36.9	2.9	36.4	3.1	36.5	3.1	36.1	3.4
2	3	572	35.8	0.3	35.5	0.8	36.4	3.7	37.2	2.5	35.8	3.6	36.2	3.3

**Notes:**

1 Mycobacterium avium subsp. paratuberculosis.

2 Cycles to threshold (CT) of qPCR.

3 SD = standard deviation.

**Figure 2:**
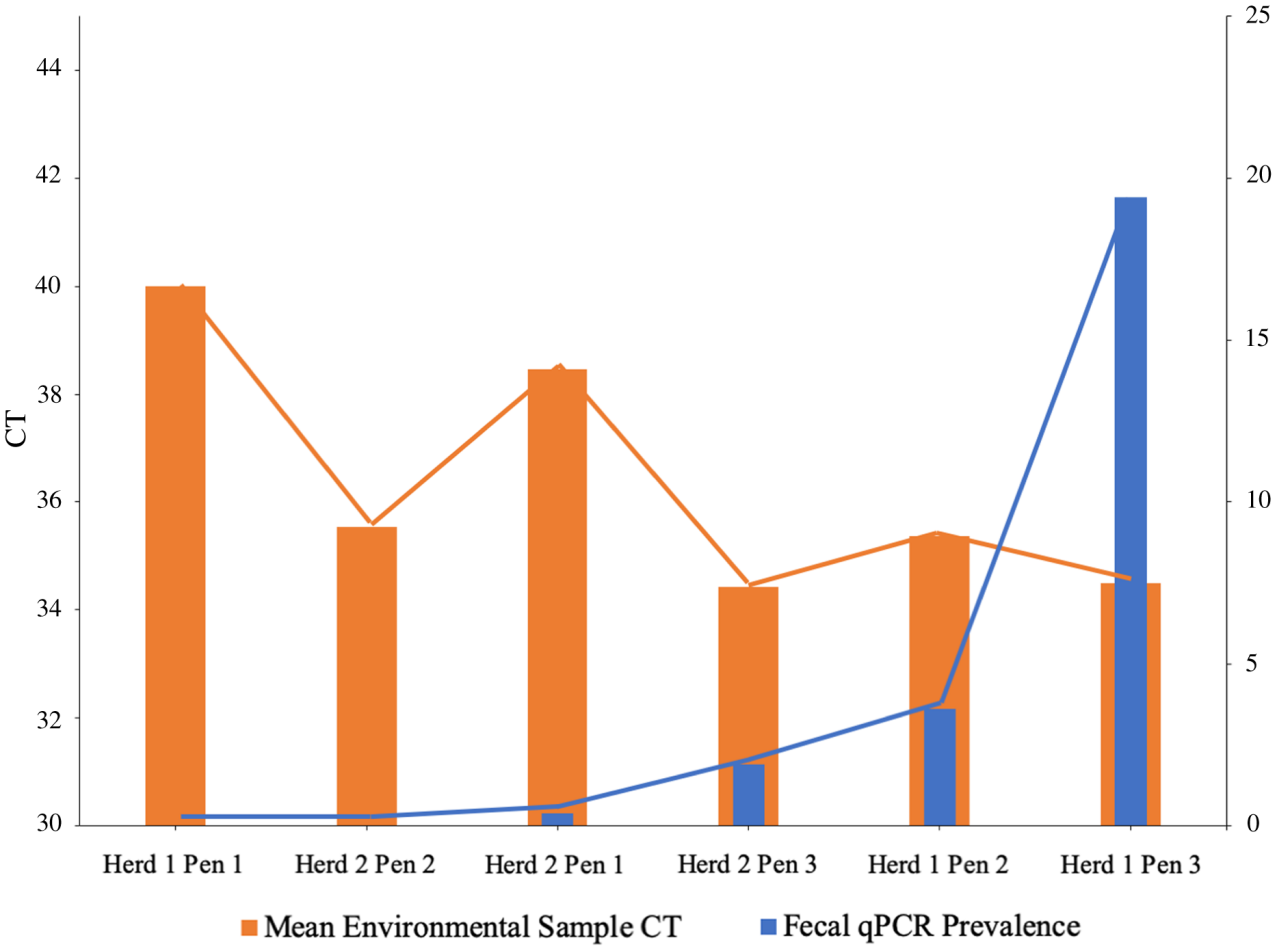
*Mycobacterium avium* subspecies *paratuberculosis* (MAP) bioburden (CT^1^) in pen environmental samples and MAP fecal shedding prevalence^2^ in drylot pens on 2 California dairies. ^1^Cycles to threshold (CT) of qPCR of environmental samples collected by two collectors from six drylot pens in two California dairies. ^2^Prevalence estimated using fecal samples collected from a stratified random sample of dairy cows and tested using qPCR for MAP.

**Figure 3:**
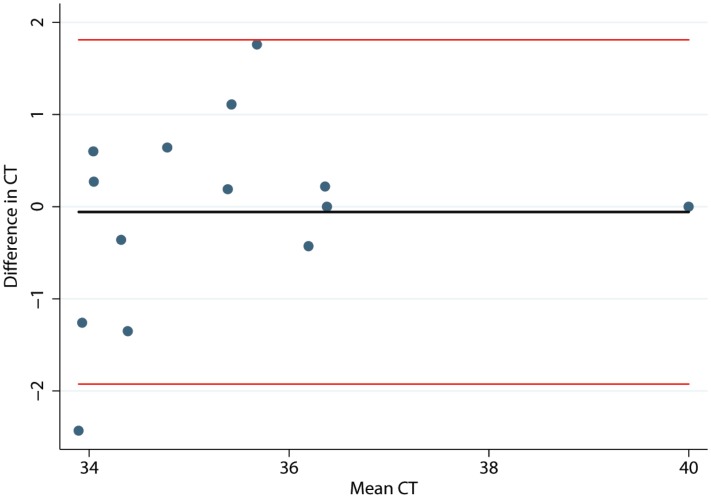
Bland and Altman plot of differences in *Mycobacterium avium* subspecies *paratuberculosis* concentration (CT) in environmental samples collected by 2 collectors against their respective means. The solid black line represents the mean of the differences in CT and the light red lines represent the 95% confidence interval.

**Figure 4:**
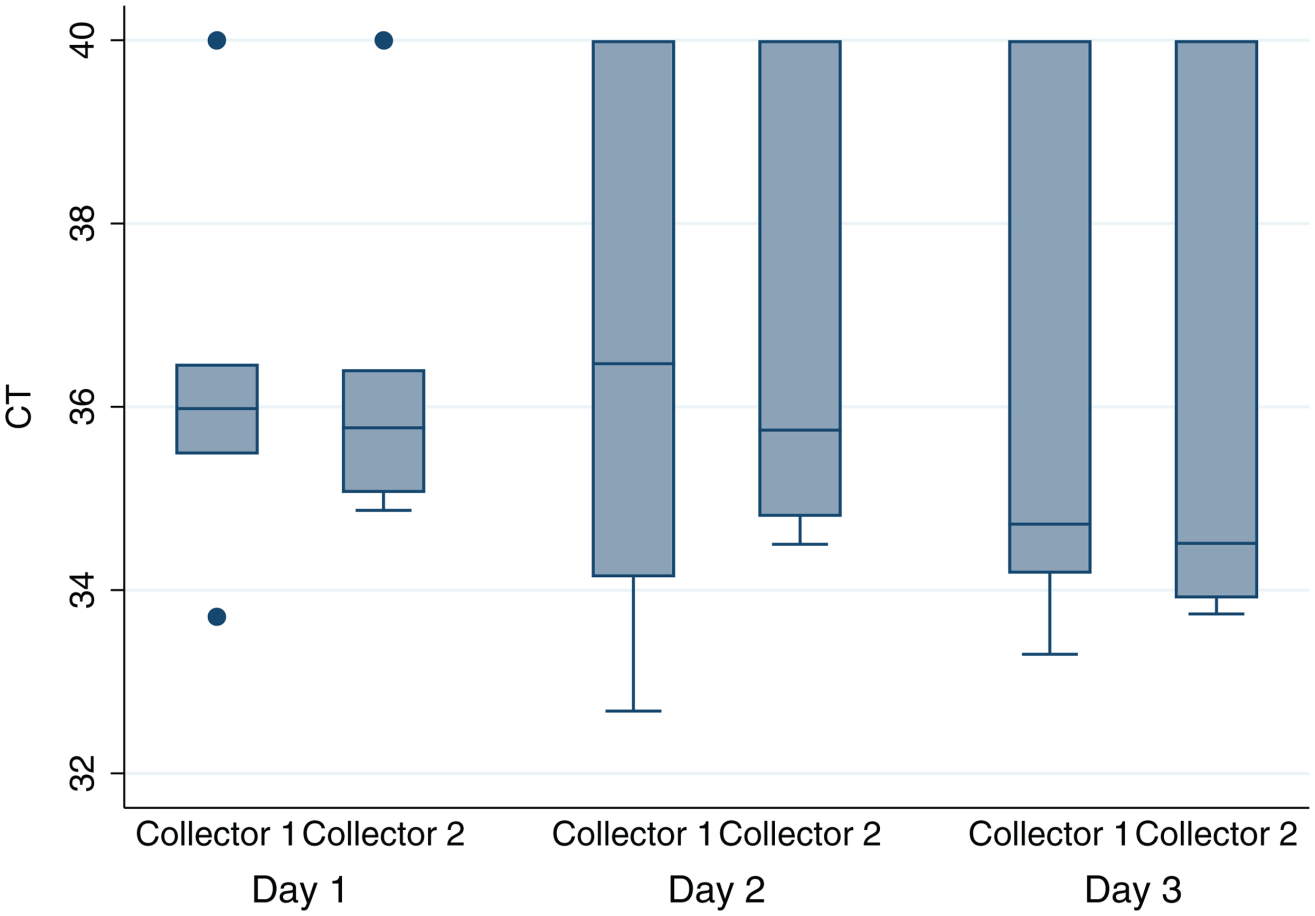
Boxplot of MAP^1^ bioburden as measured by qPCR cycles-to-threshold (CT) for environmental samples collected by two collectors from six drylot pens on two California dairies daily for 3 days. ^1^*Mycobacterium avium subsp. paratuberculosis*.

Histograms and normal quantile plots of the CT values showed no violation of the normality assumption. Figure 2 depicts the association between pen-level survey-adjusted MAP fecal qPCR prevalence and pen MAP bioburden, the later as measured using the mean of qPCR results of environmental samples collected over the 3 sampling days, by both collectors for each pen. The Spearman rank correlation coefficient for pen MAP fecal shedding prevalence and MAP bioburden was −0.72 (*p*-value = 0.1). Figure 3 shows the Bland and Altman plot depicting the difference in MAP qPCR results for samples collected by both collectors against their respective means. The mean of the collectors’ differences (bias) in CT values (SD) (Hayes et al., 1989) was −0.06 (0.93). All but 7 of the 18 differences between collectors (6 locations × 3 days) in CT values, were within the 95% limits of agreement between collectors (−0.52 to 0.41). Box plots of qPCR results are depicted in Fig. 4.

### Variance components models

Variability in CT values attributable to dairy, pen, collector and day as estimated by the variance components models are summarized in Table 3. Evaluation of residuals, standardized residuals and EBLUP of qPCR models indicated good model fit. The samples collected from different pens and different dairies accounted for the largest source of variability in MAP concentrations of environmental samples as estimated by qPCR (65.2%). Collector was a minor source of variability in the study outcome (<0.01%). Estimated variability attributable to sampling day was moderate for qPCR results (27.8%). The ICC for dairy, pen and day (measuring similarity in environmental sample qPCR results between collectors) was 93% for qPCR results and both the ICC for dairy, pen and collector (measuring similarity in environmental sample qPCR results between days) and the ICC for dairy and pen (measuring similarity in environmental sample qPCR results between collectors on different days) were 65% (Table 4).

**Table 3:** Variance of qPCR CT^1^ of MAP^2^ concentration in environmental samples collected from drylot pens in two dairies simultaneously by two collectors daily for 3 days.

Source	qPCR (CT)		
	Variance	SE^1^	% of total variability
Dairy	<0.01	0.01	<0.1
Pen	3.82	2.57	65.2
Collector	<0.01	<0.01	<0.1
Day	1.63	0.75	27.8
Error	0.41	0.14	7.0

**Notes:**

1 Cycles to threshold (CT) of qPCR.

2 Mycobacterium avium subsp. paratuberculosis.

3 SE = standard error.

**Table 4:** Intraclass correlation coefficients (ICC) estimates of similarity in MAP^1^ concentration of environmental samples collected from drypens in two dairies by two collectors daily for 3 days.

ICC	Estimate of similarity in samples of different:	qPCR (CT)			
		Estimate (%)	SE^2^	95% CI^3^	
				lower	upper
ICC (dairy, pen, day)	Collectors	92.9	3.9	85.4	100.0
ICC (dairy, pen, collector)	Days	65.1	18.1	29.6	100.0
ICC (dairy, pen)	Collectors, days	65.1	18.1	29.6	100.0

**Notes:**

1 Mycobacterium avium subsp. paratuberculosis.

2 SE = standard error.

3 CI = confidence interval.

## Discussion

The current reliability and repeatability study describes an environmental fecal sampling protocol for drylot dairy pens, a facility design used in a large proportion of western US dairies. Findings from the study show that environmental sampling using the standardized sampling protocol described followed by testing of samples by qPCR can reliably quantify MAP bioburden at the pen level on drylot pens even when implemented by different collectors, at different times, and up to 3 days apart. The approximately zero mean of differences between collectors in qPCR results indicated minimal bias between the study collectors. Simultaneous environmental sampling by two collectors yielded samples that were 93% similar in MAP concentrations.

In the present study, environmental samples collected at different times were similar to those collected by different collectors on different times within a 3 days period. Such good reliability may be explained by the minor variability attributable to collector which further justifies comparison of quantitative results of samples collected by different trained investigators on the same premises.

The reliability of environmental sampling over time indicated minor temporal changes in MAP concentrations in a pen which may be explained by the continuous mixing of fecal material from both MAP infected and non-infected cows by their movement in a pen. Furthermore, pen MAP bioburden was well correlated with MAP shedding prevalence at the pen level as quantified by qPCR. Hence, an investigator may use environmental sampling and qPCR testing to identify drylot pens with high MAP bioburden followed by sampling of individual cows in such pens as a means to identify high shedders for cost-effective surveillance of large dairy herds for MAP. This is similar to the findings of a study that used environmental sampling in freestall dairies (Aly et al., 2012). The choice of qPCR testing allowed us to detect the ISMap02 sequence which is specific to MAP. However, another study showed that non-MAP mycobacteria could give positive results by ISMAP02 targeting PCR which could have resulted in false positives (Park et al., 2018).

The ICC measure was used to estimate similarity in samples within a cluster using variance estimates from mixed models. Fixed effects were not considered for dairy, collector or day since it was of no importance to test the effect of specific dairies, collectors or sampling days but rather to consider the levels of these factors as random selections from their respective groups. Although the choice of a routine and a recently trained collector was not random, it was considered representative of extreme experience levels in environmental sampling.

Our study showed that as MAP shedding pen prevalence increases, the environmental bioburden also increases which agree with a previous study (Aly et al., 2012). The spearman rank correlation coefficient of −0.72 indicates good correlation. The negative value informs that between the MAP shedding pen prevalence and the environmental bioburden have an inverse association since pens with high MAP shedding prevalence are expected to have high bacteria concentration in their environmental samples and hence the observed lower CT values. As shown in Fig. 2, the highest MAP shedding pen prevalence was observed in herd 1 pen 3 which has the lowest mean environmental sample CT.

Both study dairies had drylot pens with concrete flush lanes which allowed for mixing of individual cow fecal pats and resulted in homogenous fecal slurry. Furthermore, the flush systems on both dairies were effective, given that the lanes were completely flushed from fecal slurry after operating the systems, thus reducing the chance of fecal slurry contamination by previous residents of the pens. For these reasons, our protocol for environmental sampling for the purpose of quantifying MAP bioburden produced satisfactory results. However, the sampling protocol described here may not perform equally on dairies with drylot pens that do not have hard-surfaces and effective flush systems. Moreover, the slurry was allowed to accumulate for 24 h prior to each sampling between flushing which possibly affects the reliability of sampling if the flush is run more or less frequently than once a day. Moreover, since most flush systems utilize recycled lagoon water it is imperative that flush is withheld from sampled pens for 24 h as in the current study to maintain the generalization of MAP bioburden to the pen population and not other pens. The study was designed specifically so that the environmental sampling occurred during a time of year when the water soakers were not operated. The results might be different if the soakers were spraying water into the lanes as common in summer months, thus diluting the fecal slurry. Hence, our findings may not be generalizable to all seasons of the year. Similarly, the sampling protocol may not perform as reliably on drylot dairies in regions with more rain than the San Joaquin Valley of California. Further research should be done during the summer when the soakers are operated or similarly during a rainy season. Additionally, the different production stages of cows (dry and lactating) may affect the results due to the higher dry matter content of dry cow feces which do not slurry as much as lactating cow feces due to differences in their diets.

The utility of environmental sampling in surveying large dairy herds housed in freestalls has been demonstrated previously (Aly et al., 2012). Testing all the cows in a large herd for MAP would be costly. However, testing environmental samples from each pen would help rank pens by bioburden and hence MAP shedding levels. Subsequently, testing cows in pen cohorts with the highest MAP bioburden can be cost-effective (Aly et al., 2012). Our findings show that environmental samples can be a reliable tool to identify high shedding cohorts of cows housed in open drylot pens too.

## Conclusions

In summary, environmental sampling is an efficient and inexpensive method to monitor MAP shedding by cattle in drylot pens with hard-surface lanes and flush. The study also showed that qPCR results of environmental samples can be comparable within 3 days when there is minimal to no cow movements between drylot pens and hence, can be used as part of a diagnostic strategy to identify pens with high MAP bioburden that would be candidates for follow-up testing of individual occupants of those pens.

## Supplemental Information

10.7717/peerj.8081/supp-1 Supplemental Information 1 Environmental samples qPCR results.

10.7717/peerj.8081/supp-2 Supplemental Information 2 Fecal samples qPCR results.

10.7717/peerj.8081/supp-3 Supplemental Information 3 Serum ELISA results.

## Additional Information and Declarations

### Data Availability

The following information was supplied regarding data availability: The raw measurements are available in the Supplemental Files.
